# Modeled Perfluorooctanoic Acid (PFOA) Exposure and Liver Function in a Mid-Ohio Valley Community

**DOI:** 10.1289/ehp.1510391

**Published:** 2016-03-15

**Authors:** Lyndsey A. Darrow, Alyx C. Groth, Andrea Winquist, Hyeong-Moo Shin, Scott M. Bartell, Kyle Steenland

**Affiliations:** 1 School of Community Health Sciences, University of Nevada, Reno, Reno, Nevada, USA; 2 Department of Epidemiology, and; 3 Department of Environmental Health, Emory University, Atlanta, Georgia, USA; 4 Department of Public Health Sciences, University of California, Davis, Davis, California, USA; 5 School of Social Ecology,; 6 Department of Statistics, and; 7 Department of Epidemiology, University of California, Irvine, Irvine, California, USA

## Abstract

**Background:**

Perfluorooctanoic acid (PFOA or C8) has hepatotoxic effects in animals. Cross-sectional epidemiologic studies suggest PFOA is associated with liver injury biomarkers.

**Objectives:**

We estimated associations between modeled historical PFOA exposures and liver injury biomarkers and medically validated liver disease.

**Methods:**

Participants completed surveys during 2008–2011 reporting demographic, medical, and residential history information. Self-reported liver disease, including hepatitis, fatty liver, enlarged liver and cirrhosis, was validated with healthcare providers. Alanine aminotransferase (ALT), γ-glutamyltransferase (GGT) and direct bilirubin, markers of liver toxicity, were obtained from blood samples collected in the C8 Health Project (2005–2006). Historically modeled PFOA exposure, estimated using environmental fate and transport models and participant residential histories, was analyzed in relation to liver biomarkers (n = 30,723, including 1,892 workers) and liver disease (n = 32,254, including 3,713 workers).

**Results:**

Modeled cumulative serum PFOA was positively associated with ALT levels (p for trend < 0.0001), indicating possible liver toxicity. An increase from the first to the fifth quintile of cumulative PFOA exposure was associated with a 6% increase in ALT levels (95% CI: 4, 8%) and a 16% increased odds of having above-normal ALT (95% CI: odds ratio: 1.02, 1.33%). There was no indication of association with either elevated direct bilirubin or GGT; however, PFOA was associated with decreased direct bilirubin. We observed no evidence of an effect of cumulative exposure (with or without a 10-year lag) on all liver disease (n = 647 cases), nor on enlarged liver, fatty liver, and cirrhosis only (n = 427 cases).

**Conclusion:**

Results are consistent with previous cross-sectional studies showing association between PFOA and ALT, a marker of hepatocellular damage. We did not observe evidence that PFOA increases the risk of clinically diagnosed liver disease.

**Citation:**

Darrow LA, Groth AC, Winquist A, Shin HM, Bartell SM, Steenland K. 2016. Modeled perfluorooctanoic acid (PFOA) exposure and liver function in a Mid-Ohio Valley community. Environ Health Perspect 124:1227–1233; http://dx.doi.org/10.1289/ehp.1510391

## Background

Perfluorooctanoic acid (PFOA, or C8) is a synthetic 8-carbon perfluorinated compound used in the manufacture of fluoropolymers for soil-, water-, stain-, and grease-resistant products (e.g., Teflon® nonstick cookware). Although PFOA production is being phased out in the United States (U.S. EPA 2013), PFOA is environmentally persistent and has been detected in the serum of more than 99% of the general U.S. population (Calafat et al. 2007). Estimates of half-life in humans range from 2.3 to 3.5 years (Bartell et al. 2010; Olsen et al. 2007).

Large doses of PFOA are known to cause liver enlargement in rodents and non-human primates (Butenhoff et al. 2002; Lau et al. 2007; Qazi et al. 2010), and hepatocellular adenomas in rats (Abdellatif et al. 2003). These effects are at least partially mediated by activation of the peroxisome proliferator-activated receptor-alpha (PPAR-α), a major regulator of lipid metabolism in the liver, but also involve the activation of other nuclear receptors (Bjork et al. 2011; Elcombe et al. 2010). Inflammatory cell infiltration and markers of oxidative stress in the liver in response to PFOA exposure have also been demonstrated in mice (Yang et al. 2014). There is debate as to whether PFOA exposure levels observed in human populations result in clinically relevant changes in liver function, particularly in light of tremendous differences between species in elimination of PFOA, with a much longer half-life observed in humans (Hundley et al. 2006; Lau et al. 2007; Olsen and Zobel 2007).

Relationships between PFOA exposures and liver enzymes in humans have been examined in several cross-sectional studies and in small occupational cohort studies. In the National Health and Nutrition Examination Survey (NHANES) PFOA has been associated with higher alanine aminotransferase (ALT) and γ-glutamyltransferase (GGT) levels, two markers of liver damage (Gleason et al. 2015; Lin et al. 2010). In a previous cross-sectional study conducted in the same community studied here, measured serum PFOA concentrations were positively associated with concurrently measured ALT levels and nonlinearly associated with direct bilirubin, a waste product of the normal breakdown of hemoglobin (Gallo et al. 2012). Other smaller studies, primarily in occupational settings, have shown inconsistent evidence of these associations (Costa et al. 2009; Emmett et al. 2006; Olsen and Zobel 2007; Sakr et al. 2007a, 2007b). Causal interpretation has been limited by the cross-sectional design of the majority of previous studies. We speculate that reverse causation is a concern because liver function could impact the storage or elimination of PFOA, which in turn affects measured serum concentrations. To our knowledge, there have been no prior published studies of clinically diagnosed liver disease and PFOA other than our recently published study of DuPont workers, which was based on 35 cases that showed a positive but non-significant trend of increasing non-hepatitis liver disease with increasing PFOA exposure (Steenland et al. 2015).

We studied a population living near the DuPont Washington Works chemical manufacturing plant in Parkersburg, West Virginia, where PFOA was used in the manufacture of fluoropolymers beginning in 1951 and peaking in the 1990s. Nearby community residents were exposed to varying levels of PFOA primarily through contaminated groundwater, with magnitude of exposure varying dramatically by water district and ranging from near-background levels to several orders of magnitude larger (Steenland et al. 2009). In this study we assess the relationship between modeled historical estimates of PFOA exposure and *a*) biomarkers of liver injury (ALT, GGT and direct bilirubin) measured in blood samples collected during 2005–2006 as part of the C8 Health Project (C8HP) and *b*) medically validated liver disease (primarily hepatitis, enlarged liver, fatty liver and cirrhosis).

## Methods

### Study Population

Methods for cohort recruitment and data collection have been described in detail elsewhere (Frisbee et al. 2009; Winquist et al. 2013). Most participants were recruited among 40,145 participants in the C8HP who were aged ≥ 20 years and consented to be contacted for further studies. The C8HP cross-sectional survey was conducted in 2005 and 2006 per the terms of a class action lawsuit and included people who were exposed for at least 12 months (at home, work, or school) to water in any of six districts contaminated (to various degrees) by PFOA. The analysis of liver biomarkers was conducted among 30,723 people from the C8HP (including 1,892 people who worked at the chemical plant) with available liver injury biomarker measurements and retrospective serum PFOA estimates. The analysis of incident liver disease included participants in the C8HP as well as additional workers who were recruited from a previously established occupational cohort (Leonard et al. 2008) of 6,026 people who worked at the chemical plant during 1948–2002; there were 32,254 people from these two cohorts who had completed at least one follow-up survey (administered between 2008–2010 and 2010–2011) and had retrospective serum PFOA estimates (3,713 from the occupational cohort and 28,541 community members who had not worked at the plant). An enrollment flowchart is available in the Figure S1. The follow-up surveys covered demographics, residential history, health-related behaviors, and lifetime personal history of various medical diagnoses. The present study was approved by the Emory Institutional Review Board (IRB) as part of the overall approval of the work of the C8 Science Panel, all of which were based on the same IRB-approved informed consent and interview.

### Exposure Estimation

Described in detail elsewhere, yearly estimates of PFOA concentrations in local air, surface water, and groundwater were generated using an environmental fate and transport model (Shin et al. 2011a), and these environmental concentrations were combined with self-reported residential history, drinking-water sources, and water consumption rates to estimate yearly PFOA intake rates. These rate estimates were then used as an input in a pharmacokinetic model to generate yearly PFOA serum concentration estimates for each study subject starting in 1952 or the year of birth, whichever occurred later (Shin et al. 2011b). For study participants who were workers at the DuPont plant, job and department-specific yearly PFOA serum concentration estimates were generated using an occupational exposure model based on historical serum PFOA measurements, participants’ work histories, and knowledge of plant operating processes (Woskie et al. 2012). For those with higher residential exposures than occupational exposures, the estimates of PFOA serum concentrations from the residential exposure model were used. For workers whose occupational exposures were higher, serum estimates were decayed 18% per year after a person stopped working at the plant (based on a half-life of 3.5 years) (Olsen et al. 2007), until they reached a level predicted by the residential exposure model. The Spearman’s rank correlation between serum concentration estimates and serum concentrations measured in blood samples in 2005–2006 among 30,303 people was 0.71 (Winquist et al. 2013).

For each follow-up year for each subject, we calculated a measure of cumulative serum PFOA exposure by summing all previous yearly estimates of PFOA serum concentrations (referred to as “cumulative PFOA”) in units of year × ng/mL. Liver disease was analyzed in relation to this measure of cumulative exposure. For liver biomarkers, we assessed this cumulative PFOA exposure measure as well as the estimated serum PFOA concentrations in the year of the participant’s blood draw, because current PFOA serum concentrations might acutely impact markers of liver injury and because associations with estimated serum PFOA, unlike measured serum PFOA, cannot be explained by reverse causation (Watkins et al. 2013).

### Outcome Definitions

***Liver biomarkers.*** We examined three markers of liver injury measured in 2005–2006 for the C8HP: alanine aminotransferase (ALT), γ-glutamyltransferase (GGT) and direct bilirubin (sometimes referred to as conjugated bilirubin). ALT enzymes are elevated after liver parenchymal cell injury and serve as a proxy of acute liver damage. Elevation of GGT enzymes is an early marker of cholestatic liver disorders, conditions in which the flow of bile from the liver is slowed or blocked. Elevated direct bilirubin usually signals problems with the liver, bile ducts, or gallbladder. Blood samples were collected from participants and centrifuged, aliquoted, and refrigerated before shipping on dry ice daily from each data collection site to the laboratory (Frisbee et al. 2009). ALT, GGT, and direct bilirubin were measured using a Roche/Hitachi MODULAR automated analyzer (Roche Diagnostics, Indianapolis, IN, USA) at a clinical diagnostic laboratory (LabCorp, Inc., Burlington, NC, USA).

In addition to modeling natural–log-transformed continuous measures of the liver biomarkers, we created dichotomized measures of each biomarker by specifying a threshold for high concentrations using the same cutoff values used in a previously published cross-sectional study in this community (Gallo et al. 2012): 45 IU/L in men and 34 IU/L in women for ALT (Schumann et al. 2002a), 55 IU/L in men and 38 IU/L in women for GGT (Schumann et al. 2002b), and 0.3 mg/dL in both men and women for direct bilirubin (McPherson and Pincus 2007). These cutoff values reflect upper limits of the reference ranges for each assay; measurements above these values would be considered abnormally high, and could prompt additional investigation into liver function in a clinical setting.

***Medically validated liver disease.*** On surveys administered between 2008 and 2011, participants were asked “Have you ever been told by a doctor or other health professional that you had any kind of liver condition such as Hepatitis, Cirrhosis, Fatty Liver, Enlarged Liver, or other liver condition?” A list of reported “other” liver diseases are available in the Supplemental Material, “Medical Records Abstraction.” For each reported condition, participants were asked “How old were you when you were first told that you had [liver condition]?” Participants were then asked to consent to medical record review; medical records were subsequently requested from the identified providers. In lieu of sending in the medical record documentation, physicians could submit an attestation form with the date of diagnosis of the self-reported condition(s), or a statement indicating “To the best of my knowledge, patient does not have this condition.” Trained medical record abstractors manually reviewed the full text of all medical record documentation submitted by providers. We focused on two outcome groupings: *a*) any medically validated liver disease and *b*) liver disease restricted to medically validated enlarged liver, fatty liver, or cirrhosis. Our purpose in analyzing the subcategory of fatty and enlarged liver and cirrhosis was to focus on liver conditions that might be expected to result from a toxic chemical exposure, excluding infectious hepatitis or “other liver disease” that includes a number of rare categories such as biliary obstruction and autoimmune liver disease. We excluded subjects whose self-reported liver disease was not medically validated, either because the medical record could not be obtained (*n* = 170, 9% of reported cases) or because the subject’s obtained medical record did not meet our *a priori* definition of liver disease (*n* = 783, 44% of self-reported cases). A list of liver conditions that were abstracted from medical records but did not meet our definition of liver disease (e.g., cysts, hemangiomas, primary or metastatic cancer) is available in the Supplemental Material, “Medical Records Abstraction.” We also excluded any subjects who did not self-report liver disease but whose medical record (obtained because of some other self-reported condition) indicated liver disease (*n* = 36) to avoid disproportionate inclusion of cases with comorbidities. For the enlarged liver, fatty liver, and cirrhosis outcome, we excluded anyone who reported hepatitis or “other” liver disease.

### Statistical Analysis

In linear regression models, natural–log-transformed liver function markers were analyzed in relation to estimated cumulative PFOA serum concentrations through 2005 or 2006 (depending on survey year) and estimated year-specific PFOA serum concentration in 2005 or 2006. Dichotomized measures of the liver function markers as defined above were modeled using logistic regression. Estimated serum PFOA was analyzed as a natural–log-transformed continuous variable and by quintiles; a *p*-value for trend across quintiles was obtained by including quintile as an ordinal variable in the model. Associations between PFOA and the liver biomarkers were adjusted for *a priori* covariates measured at baseline in 2005 or 2006 in the C8HP (parameterized as shown in Table 1 except for age): age (included as 5-year age categories), sex, body mass index (BMI), alcohol consumption, race, regular exercise, smoking status, education, household income, fasting status, history of working at DuPont plant, and insulin resistance. Insulin resistance, a predictor of liver damage, was defined using the homeostasis model assessment index (HOMA-IR: the product of basal glucose and insulin levels divided by 2.25) (Matthews et al. 1985). Models were similar to those of Gallo et al. (2012) to maximize comparability. We did not include high cholesterol or hypertension as potential confounders given these conditions may be downstream effects of the exposure (Winquist and Steenland 2014).

**Table 1:** Characteristics measured at enrollment in the C8HP (2005 or 2006) of mid-Ohio residents included in liver biomarkers study population (*n *= 30,723).

Characteristic	*N* (%)	Median PFOA^*a*^ (ng/mL)
Age (years)
20–29	4,442 (14)	11.9
30–39	5,105 (17)	12.8
40–49	6,765 (22)	14.8
50–59	6,716 (22)	19.4
60–69	4,766 (16)	25.7
70+	2,929 (10)	20.0
Sex
Male	13,658 (44)	17.1
Female	17,065 (56)	16.0
Body mass index (BMI)
Underweight (below 18.5)	386 (1)	23.0
Normal weight (18.5–24.9)	8,423 (27)	19.5
Overweight (25.0–29.9)	10,730 (35)	17.6
Obese, class I (30.0–34.9)	6,377 (21)	14.2
Obese, class II (35.0–39.9)	2,681 (9)	12.5
Obese, class III (40+)	1,835 (6)	10.8
Missing	291 (1)
Alcohol consumption
None	15,797 (51)	15.3
< 1 drink/month	5,147 (17)	15.9
< 1 drink/week	3,329 (11)	19.0
Few drinks/week	4,003 (13)	18.2
1–3 drinks/day	1,007 (3)	23.4
> 3 drinks/day	389 (1)	20.2
Missing	1,051 (3)
Race
White	29,767 (97)	16.4
Other	786 (3)	18.5
Missing	278 (< 1)
Regular exercise
Yes	10,017 (33)	19.3
No	20,706 (67)	15.4
Smoking status
Never	15,056 (49)	17.2
Former	7,912 (26)	16.3
Current < 10 cigarettes/day	1,072 (3)	15.1
Current 10–19 cigarettes/day	4,076 (13)	14.5
Current 20+ cigarettes/day	1,820 (6)	15.4
Missing	787 (3)
Education
< 12 years	3,138 (10)	14.0
High school diploma or GED	12,590 (41)	16.5
Some college	10,100 (33)	17.1
Bachelor degree +	4,748 (15)	18.1
Missing	147 (< 1)
Household income [US$/year]		
≤ 10,000	2,455 (8)	12.9
10,001–20,000	4,097 (13)	14.2
20,001–30,000	4,415 (14)	15.1
30,001–40,000	3,997 (13)	16.6
40,001–50,000	3,301 (11)	16.3
50,001–60,000	2,786 (9)	18.0
60,001–70,000	2,207 (7)	22.9
> 70,000	4,583 (15)	23.4
Missing	2,882 (9)	
Fasting status
Fasting before exam	13,087 (43)	16.2
Not fasting before exam	17,121 (56)	16.9
Missing	515 (2)	
Worker at plant
Ever	1,892 (6)	93.3
Never	28,831 (94)	14.8
ALT (IU/L)^*b*^
Mean ± SD	26 ± 19	
≤ 45 (male), ≤ 34 (female)	27,252 (89)	16.6
> 45 (male), > 34 (female)	3,471 (11)	16.0
GGT (IU/L)^*b*^
Mean ± SD	31 ± 45
≤ 55 (male), ≤ 38 (female)	26,551 (86)	16.7
> 55 (male), > 38 (female)	4,172 (14)	15.3
Direct bilirubin (mg/dL)^*b*^
Mean ± SD	0.12 ± 0.10	
≤ 0.3	30,341 (99)	16.5
> 0.3	382 (1)	16.2
Insulin resistance (HOMA-IR) [mean ± SD]	1,167 ± 1,894
^***a***^Estimated serum PFOA in the year of enrollment (2005 or 2006). ^***b***^ALT, GGT, and direct bilirubin cutoff values are consistent with Gallo et al. (2012) and reflect upper limits of the reference ranges for each assay.		

Associations between liver disease and estimated serum PFOA were examined in a survival analysis using stratified Cox proportional hazard models with age as the time scale, time-varying cumulative serum PFOA as a predictor, and stratified by birth year to control for any birth cohort trends. Cox model analyses started at the later of age 20 (to restrict to adult disease) or the subject’s age in 1952 (the year after PFOA production started at the plant) and included follow-up until 2011 at the latest, depending on the participant’s birth date, death date and timing of final interview (between 2008 and 2011). We also conducted a prospective subanalysis limited to participants who had not reported liver disease prior to the time of the C8HP in 2005/2006. In all analyses, those participants missing a diagnosis age for liver disease were excluded. We also excluded people born before 1920 (*n* = 173) because of uncertain reliability of disease self-reporting in this group. For liver disease models, quintiles were defined using the distribution of exposure estimates among cases in the year of diagnosis to maximize power. Cox models controlled for a mix of time-varying and constant covariates identified *a priori* and measured in the C8HP and follow-up interviews including: sex, years of schooling (constant; < 12 years, high school diploma/GED, some college, or bachelor’s degree or higher), race (white vs. nonwhite), smoking (time-varying; current, former, none), regular alcohol consumption (time-varying; current, former, none), BMI (at time of first study survey; underweight, normal, overweight, obese). To account for induction and latency of liver disease, in secondary analyses we also assessed cumulative PFOA exposure at a 10-year lag in relation to diagnosis of liver disease.

We investigated whether associations between PFOA and liver outcomes differed between *a*) individuals with and without a history of working at the plant *b*) men and women *c*) age < 50 years and age ≥ 50 years. Stratified analyses were conducted so that covariate effects were also estimated separately within each subgroup. Heterogeneity between strata-specific regression coefficients was tested by dividing the difference in coefficients by the square root of the sum of the variances and computing a *Z*-statistic. All analyses were conducted using SAS version 9.2 (SAS Institute, Cary, NC).

## Results

Table 1 shows the characteristics of the study population with available liver function biomarkers and historical serum concentrations as well as the median estimated serum PFOA concentration in the year of survey (2005–2006) for each characteristic. The study population for liver disease was largely composed of the same people described in Table 1, but included additional workers (total workers = 3,713) who had information on liver disease but not liver biomarker measurements (because they had not participated in the C8HP). Overall, the median estimated PFOA serum concentration in 2005–2006 was 16.5 ng/mL and ranged from 2.6 to 3559 ng/mL. The modeled cumulative serum PFOA concentration and the modeled serum PFOA concentration in 2005–2006 were highly correlated (Spearman *r* = 0.86).

### Liver Biomarkers

Table S1 shows the results of linear regression models for log-transformed liver biomarkers as a function of log-transformed cumulative serum PFOA for three models containing different nested subsets of covariates. Because results were almost identical between models including and excluding control for household income (missing for approximately 10% of participants), all subsequent models did not include household income as a covariate to maximize use of the data. The other *a priori* covariates were retained in final models because they had few missing observations and/or had a meaningful impact on estimated associations (see Table S1); only observations with complete data on these covariates were included in final models (*n* = 28,047). As shown in Table 2, the continuous (natural log transformed) measure of both cumulative and 2005/2006 modeled serum PFOA concentrations were associated with increased ALT and decreased direct bilirubin (*p* < 0.05), but neither exposure measure was associated with GGT. Assessment of PFOA by quintiles showed a monotonic increase in log-transformed ALT across quintiles of both metrics of PFOA (*p* < 0.05 for all except quintile 2 vs. quintile 1 for the 2005/2006 PFOA measure); in contrast the negative association between PFOA and log-transformed direct bilirubin was largely isolated to the fifth quintile (compared to the first). Quintile analyses also showed little evidence of an association with GGT; point estimates were in the positive direction but not statistically significant and did not show a monotonic increase across quintiles. Moving from the first to the fifth quintile of cumulative PFOA exposure was associated with an estimated 6% increase in ALT level [calculated as [exp(β)–1] × 100; 95% confidence interval (CI): 4, 8%]; this corresponds to an increase of 1.6 IU/L for an individual starting at the average ALT level of 26 IU/L, or an increase of 3.3 IU/L for an individual starting at 55 IU/L (the 95th percentile of ALT in our data).

**Table 2:** Linear regression coefficients for ln-transformed liver function biomarkers per unit increase and by quintiles of estimated cumulative and year-specific serum PFOA in 2005–2006.

Exposure parameter	Cumulative PFOA (ln y-ng/mL)			2005/2006 PFOA (ln ng/mL)		
	ALT	GGT	Direct bilirubin	ALT	GGT	Direct bilirubin
Continuous	0.012 (0.008, 0.016)	0.003 (–0.003, 0.008)	–0.005 (–0.008, –0.002)	0.012 (0.009, 0.016)	0.003 (–0.002, 0.008)	–0.006 (–0.009, –0.003)
Quintile 1	Reference	Reference	Reference	Reference	Reference	Reference
Quintile 2	0.023 (0.006, 0.040)	0.009 (–0.014, 0.031)	0.012 (–0.002, 0.026)	0.001 (–0.016, 0.018)	0.004 (–0.018, 0.026)	0.006 (–0.008, 0.019)
Quintile 3	0.035 (0.018, 0.052)	0.025 (0.003, 0.047)	–0.003 (–0.017, 0.011)	0.023 (0.007, 0.040)	0.014 (–0.008, 0.036)	0.003 (–0.011, 0.017)
Quintile 4	0.039 (0.022, 0.056)	0.011 (–0.011, 0.033)	–0.007 (–0.021, 0.007)	0.036 (0.019, 0.053)	0.015 (–0.007, 0.038)	–0.008 (–0.022, 0.006)
Quintile 5	0.058 (0.040, 0.076)	0.020 (–0.004, 0.044)	–0.017 (–0.032, –0.001)	0.048 (0.031, 0.066)	0.013 (–0.010, 0.036)	–0.018 (–0.033, –0.004)
Trend^*a*^	< 0.0001	0.1021	0.0029	< 0.0001	0.1552	0.0036
Note: The linear regression coefficients were adjusted for age, sex, BMI, alcohol consumption, regular exercise, smoking status, education, insulin resistance, fasting status, history of working at DuPont plant, and race. The percentage change in liver function biomarkers for a given change in PFOA can be calculated as [exp(β)–1] × 100, where β is the linear regression coefficient. For continuous ln PFOA, this would represent the percent change in the biomarker for a 1 ln unit increase in PFOA; for quintiles analysis, this would represent the percent change in the biomarker for a change in PFOA from quintile 1 to the specified quintile. The quintiles for estimated cumulative serum PFOA (y-ng/mL) were Q1 = 50.3–< 191.2; Q2 = 191.2–< 311.3; Q3 = 311.3–< 794.1; Q4 = 794.1–< 3997.6; Q5 = 3997.6–205667.3; Quintiles for estimated serum PFOA (ng/mL) in 2005–2006: Q1 = 2.6–< 5.8; Q2 = 5.8–< 11.4; Q3 = 11.4–< 26.7; Q4 = 26.7–< 81.5; Q5 = 81.5–3558.8. ^***a***^*p*-Value for ordinal quintile variable.						

**Figure 1:**
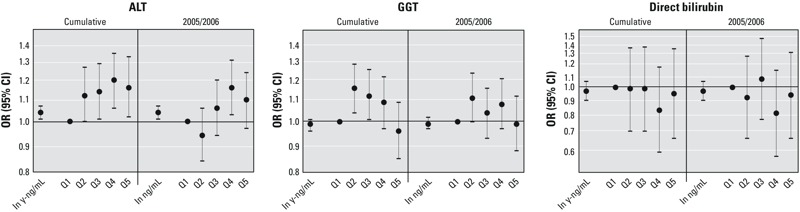
Odds ratios and 95% CIs for above normal ALT, GGT, and direct bilirubin per log increase and by quintile of cumulative and 2005/2006 year-specific modeled PFOA serum concentration (numeric results provided in Table S2).

As shown in Table 1, 11% of the study population was classified as having above normal ALT levels, 14% had above normal GGT, and 1% had above normal direct bilirubin. Figure 1 shows the results of logistic regression models for above normal ALT, GGT, and direct bilirubin as a function of estimated 2005–2006 cumulative and year-specific PFOA serum concentrations (numerical results presented in Table S2). Both metrics of PFOA were associated with high ALT levels (*p* < 0.05); the odds ratio (OR) for above normal ALT per unit increase in either cumulative (ln y-ng/mL) or year-specific PFOA (ln ng/mL) was 1.04 (95% CI: 1.01, 1.07). There was evidence of an increasing trend in the odds of having high ALT across PFOA quintiles (*p* < 0.01), with the highest ORs observed in the fourth quintile compared to the first (cumulative PFOA OR = 1.20, 95% CI: 1.06, 1.35; 2005/2006 PFOA OR = 1.16, 95% CI: 1.03, 1.31). There was little evidence for an association with above normal GGT or direct bilirubin; however, the second and third quintiles of cumulative PFOA exposure had elevated odds of high GGT relative to the first quintile (*p* < 0.05).

Analyses stratified by sex, age (< 50 and ≥ 50 years) and history of working at the plant provided no evidence of effect modification by these factors (*p* > 0.05 for interaction, similar point estimates across groups, see Table S3). Notably both metrics of PFOA were associated with higher ALT in all subgroups assessed, but the workers stratum only included 1,681 people and was not statistically significant.

### Liver Disease

There were 647 medically validated cases of liver disease included in the analysis, including fatty liver (*n* = 393), hepatitis (*n* = 157), cirrhosis (*n* = 66), enlarged liver (*n* = 44), and other liver disease (*n* = 48). Numbers of cases included in the enlarged liver, fatty liver, and cirrhosis outcome group were smaller than in the analysis of all liver disease because participants that also were diagnosed with hepatitis or other liver disease were excluded. Average age at diagnosis was 46. Table 3 presents hazard ratios for any liver disease and for liver disease restricted to enlarged liver, fatty liver and cirrhosis for cumulative PFOA with and without a 10-year lag. Table S4 shows results for the same models limited to participants who had no liver disease diagnosis prior to the C8HP and followed prospectively (included 266 cases of all liver disease and 209 cases of enlarged liver, fatty liver and cirrhosis). There was little evidence of increased risk of liver disease with PFOA exposure in any of the analyses; point estimates were mostly below the null and none of the CIs excluded the null.

**Table 3:** Hazard ratios and 95% CIs for cumulative PFOA and liver disease in the full cohort (*n *= 31,571).

Exposure parameter	Any liver disease^*a*^ (647 cases)		Enlarged liver, fatty liver, cirrhosis^*b*^ (427 cases)	
	No lag	10-year lag	No lag	10-year lag
ln y-ng/mL	0.97 (0.92, 1.03)	0.98 (0.93, 1.04)	0.97 (0.91, 1.04)	1.00 (0.94, 1.07)
Quintile 1	Ref	Ref	Ref	Ref
Quintile 2	1.19 (0.88, 1.59)	1.15 (0.81, 1.63)	0.90 (0.65, 1.25)	1.04 (0.72, 1.50)
Quintile 3	1.08 (0.81, 1.45)	1.08 (0.76, 1.54)	0.83 (0.60, 1.15)	0.91 (0.64, 1.31)
Quintile 4	1.04 (0.78, 1.40)	0.90 (0.63, 1.28)	0.75 (0.54, 1.03)	0.84 (0.59, 1.21)
Quintile 5	0.95 (0.70, 1.27)	0.99 (0.70, 1.42)	0.83 (0.60, 1.16)	0.87 (0.61, 1.25)
Note: The hazard ratios were adjusted for sex, race, education level, smoking status (current, former, none), alcohol consumption (current, former, none), BMI at time of survey, birth year (stratified). ^***a***^Includes hepatitis (*n *= 157), enlarged liver (*n *= 44), fatty liver (*n *= 393), cirrhosis (*n *= 66), and other (*n *= 48). ^***b***^Includes enlarged liver (*n *= 37), fatty liver (*n *= 363), and cirrhosis (*n *= 45); excludes cases with co-morbid hepatitis or “other” liver diagnosis.				

**Figure 2:**
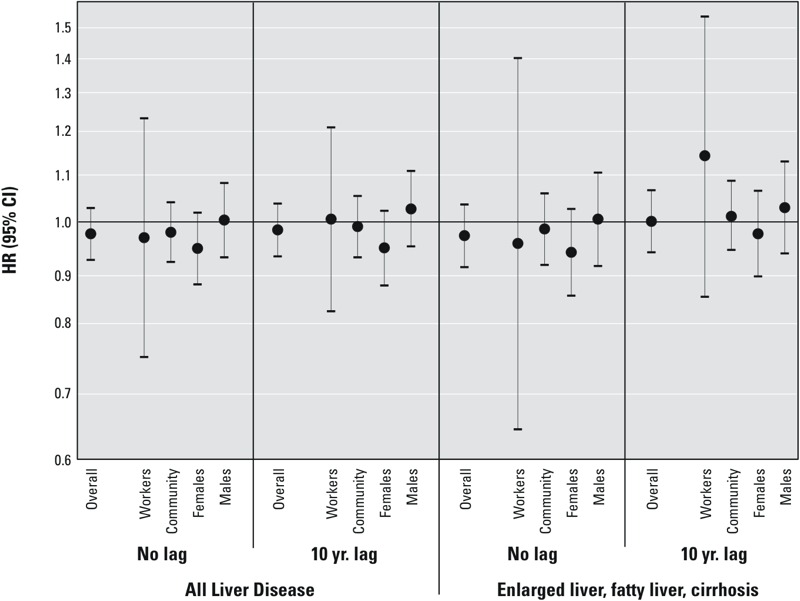
Hazard ratios (and 95% CIs) per unit of log cumulative serum PFOA for all liver disease (n = 647 cases) and for cases restricted to enlarged liver, fatty liver, and cirrhosis without co-morbid hepatitis or “other liver disease” diagnosis (n = 427 cases) stratified by sex and history of working at the DuPont plant (numeric results provided in Table S5).

In Figure 2, we present hazard ratios per natural log y-ng/mL increase in cumulative serum PFOA (with and without a 10-year lag) stratified by sex and history of working at the DuPont plant (numerical results are presented in the Table S5). Consistent with the overall analyses, these stratified analyses showed little evidence of association between PFOA and liver disease. The only elevated hazard ratio (HR) observed was for exposure at a 10-year lag and enlarged liver, fatty liver, and cirrhosis among workers (HR = 1.15; 95% CI: 0.85, 1.55), which was based on only 36 cases.

## Discussion

In this large cohort study with a wide range of exposure levels, we observed associations between modeled serum levels of PFOA and increased ALT and decreased bilirubin, but little evidence of association with GGT. We did not observe evidence that associations between PFOA and biomarkers of liver injury translate into increased risk of liver disease, including hepatitis, enlarged liver, fatty liver and cirrhosis. These associations are consistent with the conclusions of the C8 Science Panel in their probable link report for liver disease (C8 Science Panel 2012).

The association between PFOA exposure and elevated ALT, a proxy for hepatocellular injury, was consistent across analyses, being observed for ALT considered continuously or as a dichotomous outcome and for PFOA considered continuously and in quintiles for both cumulative and year-specific serum PFOA. These findings are consistent with several previous cross-sectional studies which measured serum levels of PFOA concurrently with liver function biomarkers (Gallo et al. 2012; Gleason et al. 2015; Lin et al. 2010). The potential for reverse causality or for elevated ALT and elevated serum PFOA to be downstream effects of the same underlying mechanism leads to limited causal interpretation for previous studies. For example, liver injury resulting in higher circulating ALT levels could also plausibly affect storage and excretion of PFOA driving a correlation between measured serum concentrations of PFOA and ALT, similar to the proposed explanation for cross-sectional associations between measured serum concentrations of PFOA and glomerular filtration rate (Watkins et al. 2013). Although this is of greatest concern in cross-sectional studies, this would be possible even in a prospective study if subtle pharmacokinetic differences between people drive differences in both biomarkers of exposure and liver damage (Longnecker 2006). Our study provides important complementary evidence to findings from previous studies because our external measure of PFOA dose, modeled through fate and transport from the plant and residential history in water districts contaminated to varying degrees, could not be affected by differences in pharmacokinetics between individuals.

PFOA exposure in the fifth quintile was associated with an estimated 6% increase in ALT levels relative to the first quintile (95% CI: 4, 8%) and an estimated 16% higher odds of having abnormally high ALT levels (95% CI: OR: 1.02, 1.33). The magnitude of these associations was slightly lower than previously reported in a cross-sectional study in this community using measured PFOA levels (Gallo et al. 2012); it is possible that measurement error in model-based exposure estimates attenuated associations or that previously reported cross-sectional results were partially affected by the bias related to variability in pharmacokinetics described above. Previous cross-sectional studies using data from NHANES found stronger associations between serum levels of PFOA and ALT, estimating a 1.86 unit increase in ALT per unit increase in natural log PFOA (ng/mL) (Lin et al. 2010); and when modeling log-transformed ALT, a unit increase in log PFOA (ng/mL) predicted a 3.8% increase in ALT (Gleason et al. 2015). In our population we observed a more modest 1.2% (95% CI: 0.8%, 1.6%) increase in ALT per unit increase in log PFOA (estimated serum ng/mL in 2005–2006, Table 2) which corresponds to a 0.31 unit increase from the mean ALT value of 26 IU/L in our study. However, we note the dramatic differences in exposure distribution between our study and NHANES, with 75% of U.S. adults captured by the bottom quintile of exposures in our study population (Calafat et al. 2007). A previous study among workers of the Washington Works plant with an even wider range of exposure than our population (median serum PFOA = 189 ng/mL) predicted a 2.3% increase in ALT per 1,000 ng/mL increase in PFOA (*p* = 0.124) (Sakr et al. 2007a). It is possible that the dose-response is attenuated at higher doses, which would be consistent with our results for ALT shown in Figure 2 and the stronger associations observed in populations with lower exposures.

The sources and magnitude of exposure to PFOA in the general population differ from our study population, who were largely exposed through ingestion of contaminated water. The differences in PFOA exposure sources between studies in the Ohio River Valley and the general U.S. population also suggests results are less likely to be driven by the same unmeasured or poorly measured confounder. That is, the population characteristics and behaviors associated with PFOA exposure, factors which could also be associated with liver function, differ between study populations. As one example, BMI and PFOA exposure are strongly negatively correlated in our study but slightly positively correlated in NHANES (Lin et al. 2010). Consistent associations between PFOA and ALT from study contexts with different exposure sources enhance causal interpretation.

We also observed an inverse association between PFOA and direct bilirubin, with a 0.5% decrease in direct bilirubin per log increase in PFOA. Evidence of this association in the literature is inconsistent (Costa et al. 2009; Emmett et al. 2006; Lin et al. 2010; Olsen and Zobel 2007; Sakr et al. 2007a), and comparisons between studies are complicated by differences in bilirubin measure, direct (conjugated) or total. There is evidence that perfluorinated compounds enhance destruction of bilirubin through an effect on fatty acid metabolizing CYP enzyme, (Costa et al. 2009; Pons et al. 2003). The public health implications of a decreasing effect of PFOA on bilirubin are unclear, as health concerns are typically driven by high levels. An alternative explanation for the observed inverse association is residual confounding; several risk factors for liver damage were inversely associated with PFOA in our study (e.g., obesity). If present, such confounding might also suggest that associations between PFOA and ALT and GGT are underestimated in our study. Our results differ from several previous studies that show positive associations between PFOA and GGT (Gleason et al. 2015; Lin et al. 2010; Sakr et al. 2007a). Random error in the liver biomarkers also may have obscured true associations. Although effects of PFOA would likely be chronic given its long half-life, there is considerable short-term within-person variability in the liver biomarkers (Dufour et al. 2000). Nonetheless, we note that ALT, GGT, and bilirubin are widely used in clinical practice to detect liver problems.

A major contribution of our study is the assessment of PFOA in relation to incidence of medically validated liver disease. Liver disease has been a health end point of concern for PFOA in humans based on animal studies showing that PFOA is stored in the liver and causes enlarged liver, yet almost no data are available to address this study question in human populations. To our knowledge the only previous study to examine PFOA in relation to liver disease was in an occupational subset of our study population limited by small numbers of cases (Steenland et al. 2015). In a cohort of more than 30,000 people, we identified 647 cases of medically validated liver disease, primarily fatty liver and hepatitis. Liver disease had some of the lowest rates of medical validation for diseases studied in this cohort, with less than 60% of cases of liver disease verified among participants whose records were obtained as opposed to more than 90% for coronary artery disease and malignancies, for example (Winquist et al. 2013). Some of this was likely due to a broad interpretation of liver disease by study participants and our exclusion of cysts, hemangiomas, tumors, and other documented liver-related conditions from our outcome definition. The modeling of exposure estimates back to 1951 (or birth) allowed us to maximize the number of included disease cases, but we also conducted prospective analyses restricted to disease-free individuals at the time of enrollment in 2005–2006 and observed results that were consistent with the overall analyses.

Overall we observed little evidence that PFOA exposure increases the risk of liver disease. The only suggestion of a positive association between PFOA and liver disease was among workers using a 10-year lag for enlarged liver, fatty liver, and cirrhosis. It is possible that measurement error in our exposure estimates obscured a true association, although we note that correlation between measured serum PFOA and modeled serum PFOA in 2005–2006 was moderately high (Spearman *r* = 0.71), and we were able to observe positive associations between PFOA and ALT levels using the same exposure estimates. We also acknowledge the possibility that PFOA causes a specific type of liver disease that we were unable to identify based on our broad categories of liver disease; other major causes of liver disease (e.g., viral hepatitis, alcohol) may dominate in this population making it difficult to detect small increases in risk of a specific disease subtype due to PFOA.

In summary, using exposure estimates that are not affected by reverse causation, our results complement evidence from previous cross-sectional studies showing a modest positive relationship between PFOA and ALT levels, a marker of hepatocellular injury. However, we did not observe evidence that this liver injury translates into increased risk of liver disease.

## Supplemental Material

(374 KB) PDF
