# Supplementary material for: Modeled Perfluorooctanoic Acid (PFOA) Exposure and Liver Function in a Mid-Ohio Valley Community
Source: Environ Health Perspect. 2016 Mar 15;124(8):1227–33. doi: 10.1289/ehp.1510391 (PMC4977053; doi:10.1289/ehp.1510391)
Supplement: (374 KB) PDF [file ehp.1510391.s001.acco.pdf]

Environ Health Perspect
DOI: 10.1289/ehp.1510391

Note to readers with disabilities: *EHP* strives to ensure that all journal content is accessible to all readers. However, some figures and Supplemental Material published in *EHP* articles may not conform to 508 standards due to the complexity of the information being presented. If you need assistance accessing journal content, please contact ehp508@niehs.nih.gov. Our staff will work with you to assess and meet your accessibility needs within 3 working days.

Supplemental Material

Modeled Perfluorooctanoic Acid (PFOA) Exposure and Liver Function in a Mid-Ohio Valley Community

Lyndsey A. Darrow, Alyx C. Groth, Andrea Winquist, Hyeong-Moo Shin, Scott M. Bartell, and Kyle Steenland

Table of Contents

Medical Record Abstraction

Figure S1. Enrollment of study participants for analysis of liver function biomarkers (blue) and liver disease (orange). *Note: the 30,723 included in the liver function biomarkers cohort (blue box) and the 28,541 community participants that contribute to the orange box are both subsets of the 40,145 target population. However, the liver biomarker cohort (blue box) is not a perfect subset of the liver disease cohort (orange box) because a small number of subjects included in the liver function biomarkers analysis (measured in 2005-2006) did not complete follow-up surveys (missing reported liver disease).*

Table S1. Linear regression coefficients for ln-transformed liver function biomarkers per ln y-ng/mL increase in estimated cumulative serum PFOA concentrations.

Table S2. Odds ratios and 95% confidence intervals for abnormally high values of ALT, GGT, or direct bilirubin for estimated cumulative PFOA and estimated 2005/2006 PFOA (displayed graphically in Figure 1)

Table S3. Linear regression coefficients (95% CI) for liver function outcomes per unit increase in cumulative (ln y-ng/mL) and 2005/2006 (ln ng/mL) PFOA serum concentrations from models

stratified by sex (male, female), age (< 50 years old, ≥ 50 years old), and history of working at DuPont plant (yes, no).

Table S4. Hazard ratios and 95% confidence intervals for cumulative PFOA and liver disease among those followed prospectively from 2006 (n=30,541)

Table S5. Hazard ratios and 95% confidence intervals per ln y-ng/mL increase in cumulative PFOA and liver disease stratified by sex and history of working at the DuPont plant (displayed graphically in Figure 2 in main paper)

MEDICAL RECORD ABSTRACTION

Medically-validated liver disease included as "other liver disease"

Autoimmune Liver Disease
Biliary obstruction or cholestasis
Bilirubin elevated
Enzyme deficiency
Focal Nodular Hyperplasia
Gilbert syndrome
Hemochromatosis
Jaundice not otherwise specified
Liver enzyme abnormality
Liver failure
Portal hypertension
Portal vein thrombosis
Pre-cirrhosis
Primary Biliary Cirrhosis
Sarcoidosis
Scarring of liver
Sclerosis
Toxic damage not otherwise specified

Conditions abstracted from medical records of those who self-reported liver disease that in isolation did not meet *a priori* definition of liver disease and subsequently were excluded from analysis

Abscess
Acute intermittent hepatic porphyria (usually congenital)
Alpha-1 carrier (congenital)
Cancer (primary or metastatic)
Calcifications
Cholecystectomy
Clotting factor abnormality
Cysts
Gallbladder disease
Genetic metabolic disease
HELLP syndrome (pregnancy)
Hemangioma
Hematoma
Herniated liver
Infection, non-viral
Jaundice (neonatal)
Lesion
Lipodystrophy
Liver NOS
Liver disease in pregnancy
Mercury toxicity
Mononucleosis
Polycystic liver disease
Trauma
Tumor
Tylenol toxicity

Figure S1. Enrollment of study participants for analysis of liver function biomarkers (blue) and liver disease (orange). *Note: the 30,723 included in the liver function biomarkers cohort (blue box) and the 28,541 community participants that contribute to the orange box are both subsets of the 40,145 target population. However, the liver biomarker cohort (blue box) is not a perfect subset of the liver disease cohort (orange box) because a small number of subjects included in the liver function biomarkers analysis (measured in 2005-2006) did not complete follow-up surveys (missing reported liver disease).*

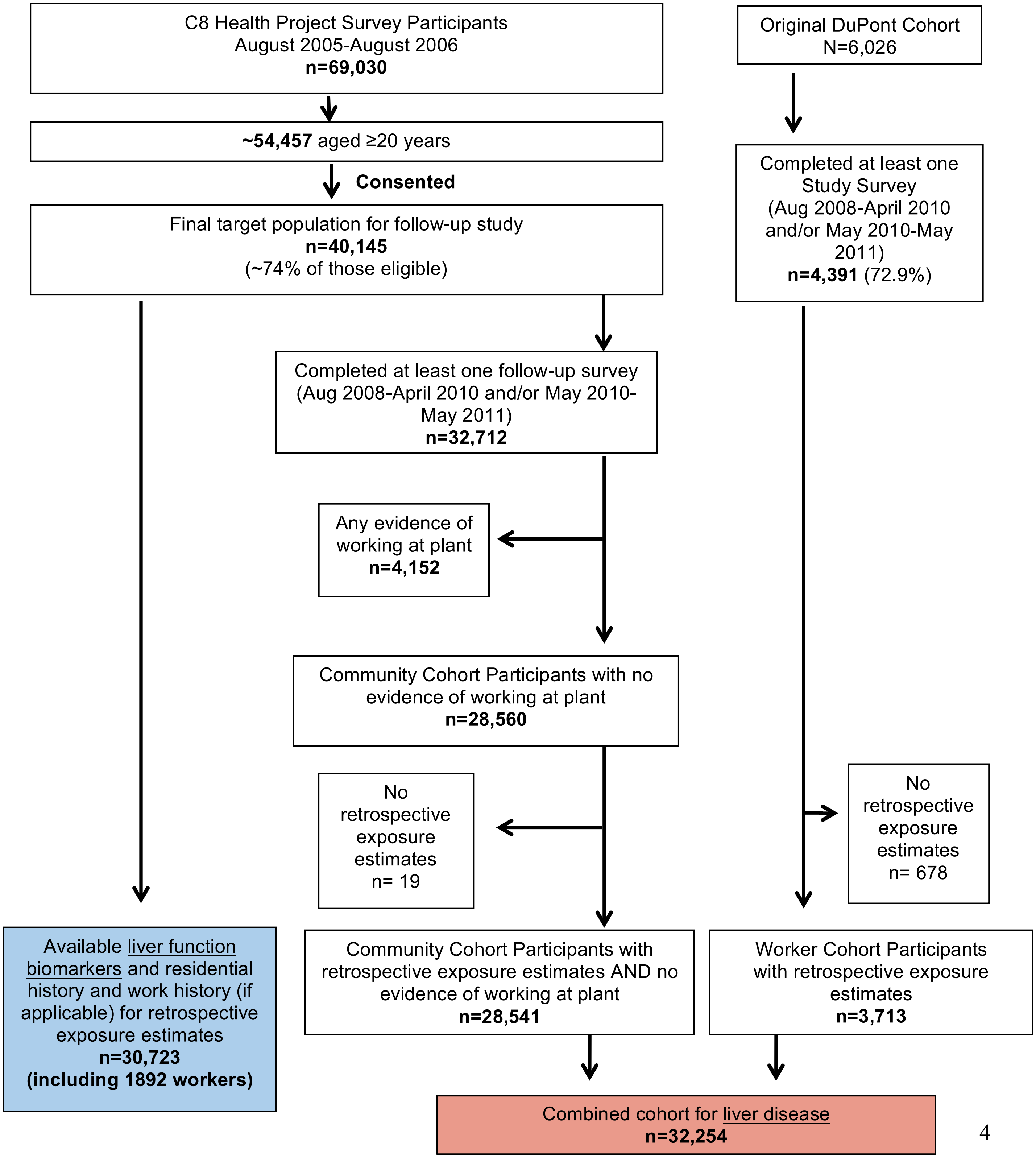

Table S1. Linear regression coefficients for ln-transformed liver function biomarkers per ln y-ng/mL increase in estimated cumulative serum PFOA concentrations.

Liver function biomarker	N	Coefficient (95% CI)	R^2	*p*-value
ln-ALT				
Model 1[a]	30,723	0.003 (-0.000, 0.007)	0.150	0.0598
Model 2[b]	28,047	0.012 (0.008, 0.016)	0.232	<0.0001
Model 3[c]	25,561	0.011 (0.007, 0.015)	0.235	<0.0001
ln-GGT				
Model 1[a]	30,723	-0.014 (-0.018, -0.009)	0.120	<0.0001
Model 2[b]	28,047	0.003 (-0.003, 0.008)	0.207	0.3078
Model 3[c]	25,561	0.004 (-0.002, 0.009)	0.208	0.1789
ln-Direct bilirubin				
Model 1[a]	30,723	0.002 (-0.001, 0.004)	0.096	0.3162
Model 2[b]	28,047	-0.005 (-0.008, -0.002)	0.150	0.0042
Model 3[c]	25,561	-0.005 (-0.009, -0.002)	0.151	0.0026

[a]Adjusted for age and sex.

[b]Adjusted for BMI, alcohol consumption, regular exercise, smoking status, education, insulin resistance, fasting status, history of working at DuPont plant, and race in addition to adjustment in model 1.

[c]Adjusted for average household income in addition to covariates in model 2.

Table S2. Odds ratios [a] and 95% confidence intervals for abnormally high values of ALT, GGT, or direct bilirubin for estimated cumulative PFOA and estimated 2005/2006 PFOA (displayed graphically in Figure 1)

	Cumulative serum PFOA			2005/2006 serum PFOA		
	ALT > 45 (♂), > 34 (♀)	GGT > 55 (♂), > 38 (♀)	Direct Bilirubin > 0.3	ALT > 45 (♂), > 34 (♀)	GGT > 55 (♂), > 38 (♀)	Direct Bilirubin > 0.3
ln ng/mL	1.04 (1.01, 1.07)	0.99 (0.96, 1.01)	0.97 (0.90, 1.05)	1.04 (1.01, 1.07)	0.99 (0.97, 1.02)	0.97 (0.90, 1.05)
Quintile 1	Reference	Reference	Reference	Reference	Reference	Reference
Quintile 2	1.12 (1.00, 1.27)	1.16 (1.04, 1.29)	0.99 (0.70, 1.38)	0.94 (0.84, 1.06)	1.11 (1.00, 1.24)	0.92 (0.66, 1.29)
Quintile 3	1.14 (1.01, 1.29)	1.12 (1.01, 1.26)	0.99 (0.70, 1.39)	1.06 (0.94, 1.20)	1.04 (0.93, 1.16)	1.07 (0.77, 1.49)
Quintile 4	1.20 (1.06, 1.35)	1.09 (0.97, 1.22)	0.83 (0.59, 1.18)	1.16 (1.03, 1.31)	1.08 (0.97, 1.21)	0.81 (0.57, 1.15)
Quintile 5	1.16 (1.02, 1.33)	0.96 (0.85, 1.09)	0.95 (0.66, 1.37)	1.10 (0.97, 1.24)	0.99 (0.88, 1.12)	0.94 (0.66, 1.33)
Trend[b]	0.0078	0.4991	0.5000	0.0055	0.7940	0.5361

[a]Adjusted for age, sex, BMI, alcohol consumption, regular exercise, smoking status, education, insulin resistance, fasting status, history of working at DuPont plant, and race. [b]*p*-value for trend across quintiles.

Quintiles for estimated cumulative serum PFOA (y-ng/mL):

Q1=50.3 - <191.2; Q2=191.2 - <311.3; Q3 = 311.3 - < 794.1; Q4: 794.1 - <3997.6; Q5=3997.6 – 205667.3

Quintiles for estimated serum PFOA in 2005-2006 (ng/mL):

Q1=2.6 - <5.8; Q2=5.8 - <11.4; Q3 = 11.4 - < 26.7; Q4: 26.7 - <81.5; Q5=81.5 – 3558.8

Table S3. Linear regression coefficients[a] (95% CI) for liver function outcomes per unit increase in cumulative (ln y-ng/mL) and 2005/2006 (ln ng/mL) PFOA serum concentrations from models stratified by sex (male, female), age (< 50 years old, ≥ 50 years old), and history of working at DuPont plant (yes, no).

	Sex		Age		Worker	
	Male (N=12,364)	Female (N=15,683)	< 50 years (N=14,969)	≥ 50 years (N=13,078)	No (N=26,366)	Yes (N=1,681)
Cumulative PFOA (ln y-ng/mL)						
ln-ALT	0.014 (0.008, 0.020)	0.010 (0.005, 0.015)	0.011 (0.006, 0.017)	0.012 (0.007, 0.017)	0.012 (0.009, 0.016)	0.010 (-0.015, 0.036)
ln-GGT	-0.002 (-0.010, 0.006)	0.006 (-0.001, 0.013)	0.003 (-0.004, 0.010)	0.002 (-0.006, 0.010)	0.003 (-0.002, 0.008)	0.016 (-0.020, 0.052)
ln-Direct Bilirubin	-0.004 (-0.009, 0.001)	-0.006 (-0.010, -0.002)	-0.005 (-0.009, -0.000)	-0.005 (-0.010, -0.000)	-0.005 (-0.008, -0.002)	-0.002 (-0.025, 0.022)
2005/2006 PFOA (ln ng/mL)						
ln-ALT	0.013 (0.007, 0.018)	0.010 (0.005, 0.015)	0.011 (0.006, 0.017)	0.012 (0.007, 0.017)	0.012 (0.008, 0.016)	0.007 (-0.011, 0.026)
ln-GGT	-0.000 (-0.008, 0.007)	0.003 (-0.004, 0.009)	0.000 (-0.007, 0.007)	0.003 (-0.004, 0.011)	0.001 (-0.004, 0.006)	0.018 (-0.009, 0.044)
ln-Direct Bilirubin	-0.006 (-0.011, -0.001)	-0.005 (-0.010, -0.001)	-0.000 (-0.010, -0.001)	-0.006 (-0.011, -0.002)	-0.006 (-0.009, -0.003)	-0.007 (-0.024, 0.010)

[a]Adjusted for age, sex, BMI, alcohol consumption, regular exercise, smoking status, education, insulin resistance, fasting status, history of working at DuPont plant, and race

Table S4. Hazard ratios[a] and 95% confidence intervals for cumulative PFOA and liver disease among those followed prospectively from 2006 (n=30,541)

	Any liver disease[b] (266 cases)		Enlarged liver, fatty liver, cirrhosis[c] (209 cases)	
	No lag	10-yr lag	No lag	10-yr lag
ln y-ng/mL	0.95 (0.87, 1.03)	0.95 (0.87, 1.04)	0.96 (0.87, 1.06)	0.98 (0.89, 1.08)
Quintile 1	Ref	Ref	Ref	Ref
Quintile 2	0.96 (0.65, 1.42)	0.99 (0.66, 1.50)	0.89 (0.57, 1.38)	1.19 (0.75, 1.88)
Quintile 3	0.96 (0.65, 1.42)	0.93 (0.62, 1.40)	0.88 (0.57, 1.38)	1.02 (0.65, 1.61)
Quintile 4	0.77 (0.52, 1.15)	0.83 (0.55, 1.24)	0.67 (0.43, 1.06)	0.94 (0.60, 1.48)
Quintile 5	0.80 (0.54, 1.20)	0.77 (0.51, 1.15)	0.86 (0.55, 1.36)	0.92 (0.58, 1.47)

[a] Adjusted for sex, race, education level, smoking status (current, former, none), alcohol consumption (current, former, none), BMI at time of survey, birth year (stratified)

[b] includes hepatitis (n=35), enlarged liver (n=20), fatty liver (n=196), cirrhosis(n=18), other (n=17)

[c] includes enlarged liver (n=17), fatty liver (n=188), cirrhosis(n=16),), excludes cases with comorbid hepatitis or “other” liver diagnosis

Table S5. Hazard ratios[a] and 95% confidence intervals per ln y-ng/mL increase in cumulative PFOA and liver disease stratified by sex and history of working at the DuPont plant (displayed graphically in Figure 2 in main paper)

	Any liver disease			Enlarged liver, fatty liver, cirrhosis		
	N cases	No lag	10-year lag	N cases	No lag	10-year lag
Overall	647	0.97 (0.92, 1.03)	0.98 (0.93, 1.04)	427	0.97 (0.91, 1.04)	1.00 (0.94, 1.07)
Female	382	0.94 (0.87, 1.01)	0.94 (0.87, 1.02)	259	0.94 (0.85, 1.03)	0.97 (0.89, 1.07)
Male	265	1.00 (0.93, 1.09)	1.03 (0.95, 1.11)	168	1.00 (0.91, 1.11)	1.03 (0.93, 1.14)
Worker	62	0.97 (0.75, 1.25)	1.00 (0.82, 1.22)	36	0.95 (0.64, 1.42)	1.15 (0.85, 1.55)
Community	585	0.98 (0.92, 1.04)	0.99 (0.93, 1.06)	391	0.98 (0.91, 1.06)	1.01 (0.94, 1.09)

[a] Adjusted for sex, race, education level, smoking status (current, former, none), alcohol consumption (current, former, none), BMI at time of survey, birth year (stratified)
